# A Koro-Like Presentation of Somatic Delusional Disorder in a Young Male With Obsessive-Compulsive Disorder: A Case Report From the United Arab Emirates

**DOI:** 10.7759/cureus.98533

**Published:** 2025-12-05

**Authors:** Mohammed Nazir, Joman Alshereida, Mohamed M Elshamy, Ali Baldo

**Affiliations:** 1 Psychiatry, Al Amal Psychiatric Hospital, Dubai, ARE

**Keywords:** cultural psychiatry, delusional disorder, koro, koro like syndrome, non endemic koro, obsessive compulsive disorder, somatic delusion

## Abstract

Koro-like syndrome (KLS) is considered a non-epidemic, culturally decontextualized variant of Koro, a culture-bound syndrome first identified in Southeast Asia. Classic Koro is marked by acute anxiety and the belief that one’s genitalia are retracting into the body, often occurring in epidemic clusters influenced by cultural and societal beliefs. In contrast, KLS arises in non-endemic settings and is typically seen in individuals with underlying psychiatric conditions - such as obsessive-compulsive disorder (OCD), psychosis, or delusional disorders - without the presence of shared cultural narratives.

We report the case of a 24-year-old Egyptian male with a history of OCD with poor insight and somatic delusional disorder who presented with persistent preoccupation about perceived testicular atrophy. He attributed this belief to a hormonal imbalance and exhibited a waddling gait to avoid pressure on his genitalia. Despite repeated medical reassurances and normal hormonal evaluations, he remained firmly convinced that his testes were undergoing atrophy.

This case highlights the diagnostic and therapeutic challenges of KLS in non-endemic settings, particularly when embedded within broader psychopathology. This underscores the importance of recognizing somatic delusions that occur outside traditional cultural frameworks and adapting treatment approaches to address the underlying psychopathology.

## Introduction

Koro-like syndrome (KLS) refers to a clinical presentation in which an individual develops a persistent, false belief that their external genitalia is atrophying, retracting, or deteriorating, often accompanied by marked anxiety or compensatory behaviors. While rooted in the classical syndrome of Koro, a culture-bound disorder first described in Southeast Asia, KLS occurs outside endemic cultural contexts and is most often associated with underlying psychiatric conditions, rather than collective belief systems [1,2].

The classic form of Koro, typically reported in China, Malaysia, Indonesia, and India, is characterized by an acute onset, intense anxiety, and a fear of imminent death due to perceived genital retraction. Patients may engage in urgent behaviors such as grasping the penis, tying it with strings, or seeking immediate medical attention. These episodes are often transient and occur in epidemic patterns, frequently precipitated by cultural beliefs or mass hysteria [2-5].

In contrast, Koro-like syndrome or secondary Koro refers to similar genital-centered preoccupations and behaviors arising in non-cultural settings, typically as a manifestation of primary psychiatric disorders. Reported associations include delusional disorder (somatic type), obsessive-compulsive disorder (OCD), schizophrenia, major depression, and body dysmorphic disorder (BDD) [3,6]. Patients with Koro-like syndrome often present with chronic symptoms, fixed somatic delusions, and engage in manual or mechanical methods to prevent perceived genital retraction [6]. Although neither Koro nor Koro-like syndromes are classified as discrete disorders in the Diagnostic and Statistical Manual of Mental Disorders, Fifth Edition (DSM-5), it is suggested to fall under “other specified obsessive-compulsive and related disorders" [7]. Recognizing such presentations outside traditional cultural contexts is therefore crucial, as they often reflect broader psychiatric comorbidities and can pose significant diagnostic and management challenges.

## Case presentation

The patient is a 24-year-old Egyptian male with a seven-year history of OCD with poor insight and somatic-type delusional disorder. He was previously enrolled in medical school but discontinued his studies due to a decline in functioning associated with the onset of psychiatric symptoms. His initial presentation included social withdrawal, poor personal hygiene, emotional blunting, and episodes of verbal and physical aggression toward family members.

Over time, his condition evolved to include both paranoid and somatic delusions. Most prominently, he developed a fixed belief that he was suffering from testicular atrophy caused by a hormonal imbalance, despite multiple normal clinical and laboratory evaluations. In addition, he reported non-specific physical complaints such as 'kidney pain' and a 'white penile discharge,' which he attributed to prescribed medications, although no organic etiology was identified. These symptoms were interpreted within the context of his broader somatic delusional framework.

Since 2023, he has experienced multiple psychiatric admissions due to treatment noncompliance and worsening symptoms. His somatic preoccupations led to marked functional impairment, including the adoption of a waddling gait, which he believed prevented further damage to his testicles. He also used various homemade and commercial herbal or nutritional “mixes” in an attempt to reverse perceived atrophy and regularly wore a supportive garment to "preserve" his testicular structure. These preoccupations significantly interfered with his academic pursuits and daily activities.

During his most recent admission, prompted by escalating aggression and bizarre behavior, the patient continued to express a fixed belief that normal walking would “squeeze and damage” his testicles. He appeared markedly anxious when discussing these concerns. On mental status examination, his affect was restricted, thought content was dominated by somatic delusions, and insight into the delusional nature of his beliefs was absent. He denied hallucinations, disorganized speech, or substance use. His obsessive-compulsive symptoms were severe, corresponding to a Y-BOCS score of approximately 32, reflecting significant distress and impairment in daily functioning [8,9].

To address persistent medication refusal, long-acting injectable aripiprazole (400 mg intramuscularly monthly) was initiated, alongside oral aripiprazole, which was gradually tapered from 20 mg to 10 mg daily. He was maintained on fluvoxamine 100 mg twice daily for OCD symptoms and propranolol 10 mg twice daily for anxiety and somatic tension. Follow-up clinic notes documented improvement in behavioral stability, reduction of intrusive obsessive thoughts, absence of aggressive outbursts, and partial improvement in insight. His OCD severity decreased from severe to moderate, with an estimated Y-BOCS score of 20, and he was able to resume routine daily activities. Plans were made to continue monthly aripiprazole injections alongside fluvoxamine and propranolol.

## Discussion

This patient presents with a chronic, non-epidemic form of KLS, accompanied by OCD with poor insight and somatic-type delusional disorder. Unlike classical Koro - typically characterized by acute onset, fear of imminent death, and rooted in cultural panic - this patient displayed a persistent and fixed belief that his testicles were shrinking due to a hormonal disturbance. His compensatory behaviors, including a waddling gait to reduce perceived pressure on the genitalia, the use of scrotal support garments, and reliance on various herbal remedies, reflect a functional adaptation to a deeply entrenched somatic delusion. 

A similar case of Koro syndrome occurring in the context of OCD was reported by Silva et al. (2016), in which genital retraction beliefs were integrated into the patient’s obsessive-compulsive symptomatology, highlighting the diagnostic overlap between Koro-like presentations and OCD with poor insight [10]. Similarly, El-Badri and Leathart (2017) reported a case of a 66-year-old man with paranoid schizophrenia and KLS, who experienced chronic penile shrinkage delusions and altered urination posture without fear of death [11]. Additionally, a case involving an 18-year-old Muslim male initially diagnosed with Koro syndrome revealed comorbid OCD during treatment. After adjusting his regimen to include sertraline 150 mg/day, clonazepam 0.5 mg three times daily, and supportive psychotherapy, his Koro-related symptoms resolved significantly within one month [12]. In comparison, our patient similarly exhibits a Koro-like presentation rooted in delusional and obsessive psychopathology, but with more pronounced functional impairment and repeated treatment nonadherence, highlighting the need for sustained and multimodal management strategies.

The distinction between Koro as a culturally specific syndrome and as a manifestation of broader, cross-cultural psychopathology remains a subject of debate. Sporadic Koro-like presentations have been documented in association with various neurological and psychiatric conditions, including brain tumors, temporal lobe epilepsy, stroke, substance use disorders, depressive illness, phobic disorders, and schizophrenia. These reports suggest that KLS may arise as a nonspecific expression of underlying psychopathology rather than as a phenomenon limited to specific cultural contexts [13]. Whereas typical Koro epidemics are marked by acute anxiety and panic in response to perceived genital retraction, Koro-like presentations tend to reflect the clinical features of the underlying psychiatric or neurological disorder [14].

Furthermore, treatment approaches for Koro and KLS vary widely depending on cultural context. In Southeast Asia and parts of Africa, management often involves traditional or folk remedies rooted in cultural beliefs, while in Western settings, psychopharmacological and psychotherapeutic interventions are typically employed [15]. Treatment strategies for secondary Koro syndrome emphasize managing the primary psychiatric disorder. In our case, antipsychotics and selective serotonin reuptake inhibitors (SSRIs) were prescribed, though poor insight and noncompliance posed challenges. Long-acting injectable aripiprazole was considered due to recurring relapses.

## Conclusions

This case illustrates a rare, culture-transcending presentation of KLS within the context of comorbid OCD and somatic delusional disorder, challenging the notion of Koro as a purely culture-bound phenomenon. It underscores the need for careful assessment of Koro-like symptoms, as they may mask underlying OCD-delusional overlap disorders. Clinically, long-acting injectable antipsychotics can be particularly useful in managing severe nonadherence in such cases. Ultimately, a multidisciplinary approach with long-term follow-up and adherence-focused strategies is essential to achieving sustained recovery.
